# Dipeptidyl Peptidase-4 Inhibitor-Associated Bullous Pemphigoid

**DOI:** 10.3389/fimmu.2019.01238

**Published:** 2019-06-04

**Authors:** Kaisa Tasanen, Outi Varpuluoma, Wataru Nishie

**Affiliations:** ^1^PEDEGO Research Unit, Department of Dermatology, Medical Research Center Oulu, Oulu University Hospital, University of Oulu, Oulu, Finland; ^2^Department of Dermatology, Hokkaido University Graduate School of Medicine, Sapporo, Japan

**Keywords:** BP180, bullous pemphigoid, CD26, collagen XVII, diabetes mellitus, DPP4, gliptins

## Abstract

Bullous pemphigoid (BP) is an organ-specific autoantibody-mediated blistering skin disease that mainly affects the elderly. Typical clinical features include the widespread blisters, often preceded by and/or associated with itchy urticarial or eczema-like lesions. BP patients have circulating autoantibodies against BP180 and/or the plakin family protein BP230 both of which are components of hemidesmosomes in basal keratinocytes. Most BP autoantibodies particularly target the epitopes within the non-collagenous NC16A domain of BP180. Clinical findings and murine models of BP have provided evidence of a pathogenic role of anti-NC16A autoantibodies. However, it is largely unknown what triggers the breakage of immunotolerance against BP180 in elderly individuals. The incidence of BP has been increased over the past two decades in several countries. Aside from aging populations, the factors behind this phenomenon are still not fully understood. Neurodegenerative diseases such as multiple sclerosis, Parkinson's disease, and certain dementias are independent risk factors for BP. Recently several case reports have described BP in patients with diabetes mellitus (DM) patients who have been treated with dipeptidyl peptidase-4 inhibitors (DPP-4i or gliptins), which are a widely used class of anti-DM drugs. The association between the use of DPP-4is, particularly vildagliptin, and BP risk has been confirmed by several epidemiological studies. Evidence suggests that cases of gliptin-associated BP in Japan display certain features that set them apart from cases of “regular” BP. These include a “non-inflammatory” phenotype, targeting by antibodies of different immunodominant BP180 epitopes, and a specific association with the human leukocyte antigen (HLA) types. However, recent studies in European populations have found no major differences between the clinical and immunological characteristics of gliptin-associated BP and “regular” BP. The DPP-4 protein (also known as CD26) is ubiquitously expressed and has multiple functions in various cell types. The different effects of the inhibition of DPP-4/CD26 activity include, for example, tissue modeling and regulation of inflammatory cells such as T lymphocytes. Although the pathomechanism of gliptin-associated BP is currently largely unknown, investigation of the unique effect of gliptins in the induction of BP may provide a novel route to better understanding of how immunotolerance against BP180 breaks down in BP.

## Clinical, Histological, and Immunological Features of BP

**Figure 1:**
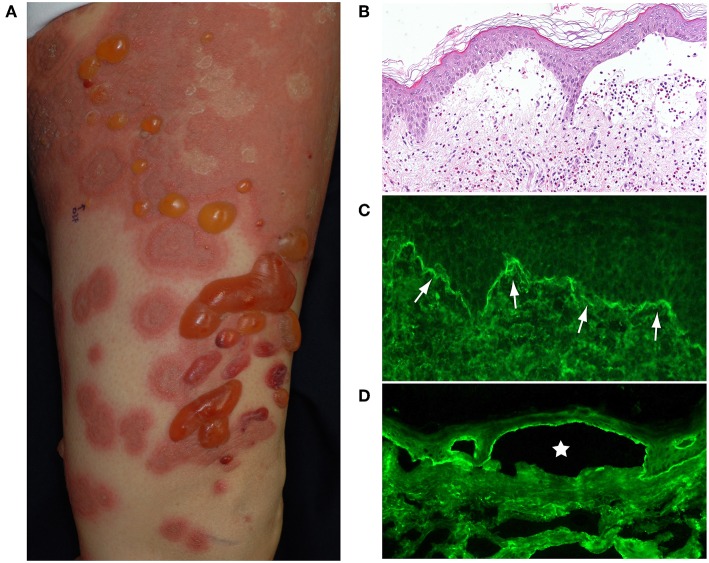
**(A)** Typical BP patient who had widespread itchy urticarial and erythematous lesions and tense blisters. **(B)** The histopathological analysis of lesional skin samples shows subepidermal blisters with numerous eosinophilic infiltrates. **(C)** Direct IF of a peri-blistering lesion reveals the linear deposition of IgG at the dermo-epidermal junction (arrows). **(D)** Indirect IF detects circulating IgG autoantibodies directing the dermo-epidermal junction of normal salt-split human skin. The IgG reacts with the roof epidermis of an artificial blister (star). This figure has been previously published in the following paper: Nishie (1). New diagnostic tool for bullous pemphigoid: full-length BP180 ELISA.

Typically, elderly BP patients have tense blisters, erosions, crusts and erythema over their entire body (Figure 1A). Before the development of blisters, BP is often preceded by a prodromal phase, characterized by severe pruritus and/or non-specific skin symptoms, which may include eczematous, urticarial, papular or excoriated lesions. As many as 20–30% of BP patients have no blistering, and this can delay or hamper correct diagnosis (2, 3).

Histopathologically, BP blisters form in the subepidermis and contain numerous eosinophilic infiltrates (Figure 1B). A diagnosis of BP is based on clinical manifestations, direct biopsy (Figure 1C) and indirect (Figure 1D) immunofluorescence (IF) microscopy on salt split human skin and serological assays [BP180-NC16A enzyme-linked immunosorbent assay (ELISA)]. The direct IF study, which is the gold standard of BP diagnostics, reveals the linear deposition of IgG and/or complement C3 at the dermal-epidermal junction of perilesional skin (Figure 1C).

## Pathogenesis of BP

**Figure 2:**
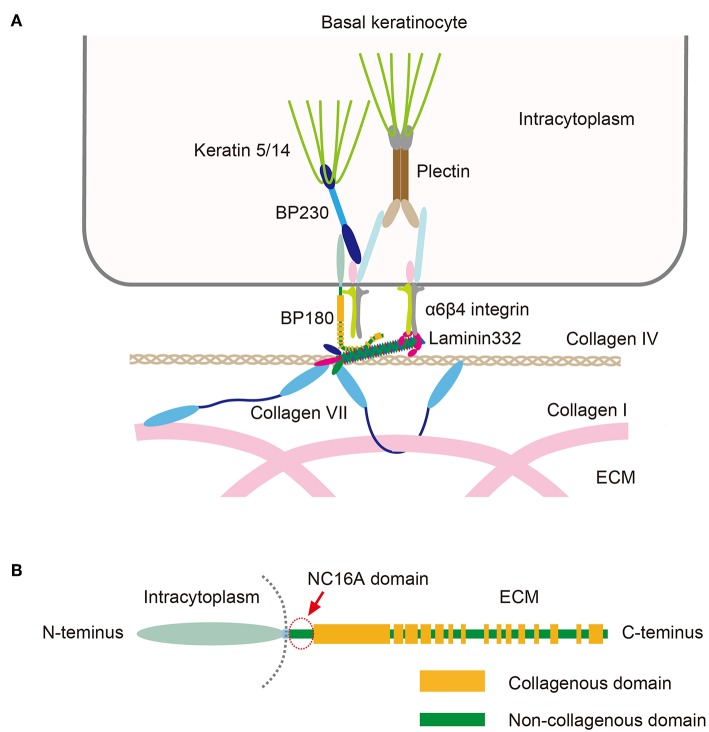
**(A)** Schematic presentation of molecules that make up the dermo-epidermal junction. **(B)** The structure of BP180. Note that the NC16A domain (arrow) is the juxtamembranous extracellular domain. This figure has been previously published in the following paper: Nishie (4). Update on the pathogenesis of bullous pemphigoid: an autoantibody-mediated blistering disease targeting collagen XVII.

BP autoantibodies target BP180 and BP230, both of which are hemidesmosomal proteins that play essential roles in maintaining stable adhesion between the epidermis and the dermis (Figure 2) (4, 5). Also known as collagen XVII, BP180 is a 180 kD type II-orientated transmembrane collagen with its amino terminus located in the cytoplasm and its carboxyl terminus in the extracellular matrix (Figure 2). BP230 is a 230 kD protein of the plakin family that is found in the cytoplasm. Around 85% of BP immunoglobulin (Ig)G autoantibodies target the juxtamembranous extracellular non-collagenous (NC16A) domain of BP180 and levels of anti-NC16A correlate well with the severity of BP (4, 5). Passive transfer of anti-BP180 NC16A domain IgG autoantibodies from BP patients induces skin fragility in transgenic mice that express human BP180 (6, 7), suggesting that the anti-NC16A autoantibodies play a central role in blister formation. In contrast, the significance of anti-BP230 autoantibodies is unclear. In addition to IgG autoantibodies, BP patients also have IgE-class autoantibodies, which are thought to be associated with the inflammatory characteristics of BP (8). The binding of BP autoantibodies to their epitopes activates complement, which leads to the degranulation of mast cells and the release of leukotrienes, TNF-α and other cytokines (4, 5). These mediators activate neutrophils and eosinophils, which produce proteolytic enzymes that are able to degrade the dermo-epidermal junction, which has the end result of blister formation (4, 5).

## Epidemiology of BP

### Incidence, Comorbidities, and Mortality

Estimates of the annual incidence of BP range between 2.5 and 21.7 cases per million persons (9–11). For example, the age-standardized incidence of BP has been reported as 14 per million person-years in both Finland and Scotland (12, 13). Several studies have reported a trend of increased incidence of BP over the last few decades, including some that controlled for the effects of an aging population (12, 14).

BP is an atypical autoimmune disease because it normally develops in the elderly; reports of the mean age at diagnosis vary between 69 and 83 years (10, 14–20). Since BP is a disease most commonly seen in elderly patients, BP is frequently accompanied by several comorbidities (21). A recent study found that 84% of subjects with BP also had at least two other chronic diseases (22). Hypertension, psoriasis and diabetes are among the most common comorbidities (22–25). Reports of associations between BP and malignancies are inconsistent. A recent meta-analysis found that BP was not associated with overall cancer morbidity, although there was an association between BP and hematological malignancies (26).

Patients with BP have a heightened risk of death. An overall 1-year mortality rate of 23.5% in patients with BP (range 6–41%) was reported in a recent systematic review and meta-analysis (27). Advanced age at BP onset, diagnostic delay and comorbid diabetes are each associated with increased 1-year mortality (19). Polypharmacy is common among BP patients and it has been associated with higher mortality: the more drugs a BP patients receives, the greater their mortality risk (17).

### Neurological Diseases Increase the Risk of BP

Several epidemiological studies indicate that neurological and neurodegenerative diseases are common in BP patients (21, 28–30). More specifically, multiple sclerosis (MS), Parkinson's disease (PD) and certain dementias, including Alzheimer's disease (AD), significantly increase an individual's risk of developing BP (28, 29). BP180 and a neural isoform of BP230 are expressed in the brain as well as in the skin (31). The strong epidemiological association between neurological disorders and BP, alongside the neuronal expression of BP autoantigens has led to the assumption that neurodegeneration or neuroinflammation could lead to the failure of self-tolerance against BP180 and BP230 and thus the development of BP (32–34). Some patients with AD, MS or PD have circulating autoantibodies against BP180, but these non-pathogenic autoantibodies do not bind to the skin and therefore do not cause cutaneous symptoms (32–34). Currently it is not known whether epitope spreading or some other mechanism or factors lie behind the high risk of BP carried by neurological patients. A recent nationwide Finnish registry study suggested that the use of some drugs that affect the nervous system may contribute to the onset of BP, but additional studies are required to confirm this association (35).

### Medication As a Risk Factor for BP

To date, more than 60 drugs have been reported to induce BP, including certain antibiotics, diuretics and other anti-hypertensive drugs, anti-TNF-α-drugs and vaccines (36). Of all drug classes, robust evidence suggests that prior use of dipeptidylpeptidase-4 inhibitors (DPP-4i or gliptins) carries the highest risk for BP. Currently there is a rapidly growing volume of publications regarding DPP-4i-associated BP (Tables 1, 2), meaning that it is now an important issue in the field.

**Table 1:** Selected case reports of gliptin-associated bullous pemphigoid.

**First author**	**Number of cases**	**DPP-4 inhibitor used (n)**	**Latency time**
Pasmatzi et al. (37)	2	Vildagliptin (2)	2 months
Skandalis et al. (38)	6	Vildagliptin (5), sitaglitpin (1)	2–13 months
Aouidad et al. (39)	3	Vildagliptin (1), sitagliptin (2)	5–6 months
Attaway et al. (40)	1	Sitagliptin (1)	12 months
Bene et al. (41)	3	Vildagliptin (3)	1–37 months
Mendonça et al. (42)	3	Vildaglitpin (2), linagliptin (1)	45 days−3 months
García et al. (43)	1	Vildagliptin	12 months
Haber et al. (44)	2	Linagliptin (2)	3–4 months
Sakai et al. (45)	1	Linagliptin (1)	9 months
Esposito et al. (46)	1	Linagliptin (1)	5 months
Yoshiji et al. (47)	5	Vildaglitpin (1), linagliptin (2), sitagliptin (1), anagliptin (1)	1–15 months
Harada et al. (48)	1	Sitagliptin (1)	3 years
Oya et al. (49)	1	Anagliptin (1)	1 month
Schaffer et al. (50)	9	Vildagliptin (4), sitagliptin (5)	5–48 months
Fania et al. (51)	5	Vildagliptin (1), sitagliptin (1), linagliptin (2), alogliptin (1)	1–8 months
Lindgren et al. (52)	10	Vildagliptin (4), sitagliptin (5), linagliptin (1)	5–24 months

**Table 2:** Epidemiological studies of gliptin-associated bullous pemphigoid.

**First author**	**Country**	**Population**	**Cases/controls, n**	**Mean age (cases), y**	**Adjusted OR**
Schaffer et al. (50)	Switzerland	Hospital data	23 (DM+BP)/170(DM)	77.6	DPP-4i: 2.48 (95% CI 0.75–8.3)
Benzaquen et al. (53)	France	Hospital data (3 hospitals)	61 (BP+DM)/122(DM)	79.1	DPP-4i: 2.64 (95% CI 1.19–5.85) Vildagliptin: 3.57(95% CI 1.07–11.84) Sitagliptin: 2.13(95% CI 0.77–5.89) Linagliptin/saxagliptin: 2.90 (95% CI 0.47–17.74)
Varpuluoma et al. (54)	Finland	Nationwide registry data	3397/12941	76.6	DPP-4i: 2.19 (95% CI 1.55–3.11) Vildagliptin: 10.4(95% CI 4.56–23.80) Sitagliptin: 1.37 (95% CI 0.93–2.01) Metformin: 1.05 (95% CI 0.88–1.24)
Kawaguchi et al. (55)	Japan	Hospital data	168 cases: DPP4i-BP 32 non-DPP4i-BP 136	79.7	NA^a^
Kridin and Bergman (56)	Israel	Hospital data	82 (BP+DM)/328 (DM)	79.1	DPP-4i: 3.16 (95% CI 1.86–5.37) Vildagliptin: 10.67 (95% CI 5.09–22.36) Linagliptin: 6.65 (95% CI 2.24–19.72) Sitagliptin: 0.42 (95% CI 0.12–1.45)
Plaquevent et al. (57)	France	Hospital data (21 hospitals). general population from reimbursement register	1787/225412	77.9	NA^b^
Lee et al. (58)	Korea	Insurance data, nationwide	670 (BP+DM)/ 670 (DM)	75.3	DPP-4i: 1.58 (95% CI 1.25–2.00) Vildagliptin: 1.81 (95% CI 1.31–2.50) Sitagliptin: 1.70 (95% CI 1.19–2.43) Linagliptin: 1.64 (95% CI 1.15–2.33) Other DPP-4is: 1.25 (95% CI 0.73–2.14)

*BP, Bullous pemphigoid; CI, Confidence interval; DM, Diabetes mellitus; DPP-4i, Dipeptidyl peptidase-4 inhibitor; OR, Odds ratio*.

a *BP incidence 0.0859% of patients receiving DPP-4is*.

b *Observed frequency of DPP-4is and vildagliptin compared to general population (6.0 vs. 3.6% and 3.3 vs. 0.7%)*.

In addition immune checkpoint inhibitors against programmed death-1 (anti-PD-1) and programmed death-ligand 1 (anti-PD-L1) have recently been added to the list of drugs associated with BP (59, 60). Anti-PD-1 and -PD-L1 medications are used to treat metastatic melanoma and other advanced malignancies. As many as 20% of treated patients develop dermatological adverse events, predominantly non-specific rashes and pruritus (60). Since 2015, there have been at least 29 case reports of an association between treatment with anti-PD-1/PD-L1 medication and BP (60). A recent retrospective study of 853 patients found BP in 0.8% of patients treated with PD-1 or PD-L1 inhibitors (59).

## DPP-4i-Associated BP

### DPP-4

The DPP-4 protein is a member of the prolyl-oligopeptidase superfamily, which between them cleave a wide range of bioactive peptides (61). DPP-4 (also known as CD26) is ubiquitously expressed in various kinds of cells, including T-lymphocytes (62). The expression of the DPP-4/CD26 protein is increased in several skin diseases, including such as T-cell lymphomas, psoriasis, lichen planus and atopic dermatitis (63). The cutaneous expression of DPP-4/CD26 was recently shown to be upregulated in BP patients, but independently of prior gliptin treatment (52).

### DPP-4 Inhibitors: A Widely Used Drug Class for the Treatment of Type II Diabetes Mellitus

Among other substrates DPP-4 degrades incretins, a group of metabolic hormones, two of which are glucagon-like peptide-1 (GLP-1) and glucose-dependent insulinotropic polypeptide (GIP) (64, 65). Inhibition of the enzymatic activity of DPP-4 prolongs the GLP-1/GIP-dependent secretion of insulin by pancreatic beta cells that is induced by increased serum glucose levels. Therefore, DPP-4is have beneficial effects on blood glucose levels, but their use does not carry a relevant risk of hypoglycemia. In addition to its effects on GLP-1 and GIP, DPP-4 is also involved in the N-terminal dipeptide cleavage of various molecules including eotaxin (CCL11), regulated on activation, normal T-cell expressed and secreted (RANTES), CCL5, CXCL9, CXCL10, and CXCL11 (64). Thus, DPP4-is may have functions other than their anti-hyperglycemic effects. It has also been reported that DPP-4is may promote regeneration following endothelial and myocardial injury, and may inhibit the development of atherosclerosis (66). Furthermore, DPP-4is may target other proteins in the DPP family, including DPP-8 and DPP-9, whose biological functions have not yet been fully elucidated (65). Since 2006, when sitagliptin became the first DPP-4i to be licensed, more than 10 other DPP-4is have been approved for clinical use.

### Case Reports of DDP-4i-Associated BP

Skandalis et al. (38) were the first researchers to describe gliptin-associated BP. They reported five cases in which BP arose following use of DPP-4i and metformin for periods lasting between 2 and 13 months. Since then, numerous case reports of DPP-4i-associated BP have been published originating from different countries and concerning all the available DPP-4i drugs (Table 1).

### Registry and Cohort Studies Showing the Association Between the Use of DPP-4i Medication and BP

There is an impressive body of epidemiological evidence showing the association between the use of DPP-4i medication and BP (Table 2). Reports from European and French pharmacovigilance databases were the first epidemiological studies to show a disproportionately high incidence of BP in patients treated with DPP-4is (43, 67). A study using reports filed in the EudraVigilance pharmacovigilance database found that BP was associated with treatment with vildagliptin, linagliptin, saxagliptin, and sitagliptin (43). Vildagliptin was the DPP-4i most commonly reported to induce BP. A similar disproportionality was also noted in a Japanese pharmacovigilance database study, particularly among patients treated with vildagliptin, linagliptin, and teneligliptin (68).

In a Swiss case-controlled study of 23 diabetic BP patients and 170 diabetic controls DPP-4i use was more frequent in the diabetic BP patients than the controls (39.1 and 33.5%, respectively) although the difference did not reach statistical significance (50). Another case-controlled study of 61 BP patients with diabetes and 122 diabetic controls found that DPP-4i use increased the risk of BP almost 3-fold, but the increase was driven by vildagliptin rather than the other DPP-4is (53). An Israeli case-controlled study of 82 BP patients also found that vildagliptin was the DPP-4i most strongly associated with BP risk, although it also found an increased risk for BP with linagliptin (56). The first nationwide registry study from Finland demonstrated a 10-fold-elevated risk of BP after the use of vildagliptin in a case-controlled setting (54). In that study combination therapy with metformin and vildagliptin or sitagliptin was associated with an increased risk of BP, but no such association was seen with metformin alone (54). Another nationwide study from Korea also found that the use of DPP-4is was associated with a significant increase in the risk of developing BP; with vildagliptin again presenting the highest risk (58). Accordingly, a recent French study found that the observed per-capita intake of DPP4is, particularly that of vildagliptin was higher in a cohort of 1,787 patients with BP than in the general population (57).

### Clinical and Immunological Characteristics of DPP-4i-Associated BP

The reported latency between the initiation of DPP-4i medication and a diagnosis of BP is generally several months, but may be more than 1 year (Table 1) (43, 53, 54, 56, 57). The long latency between the initiation of a gliptin and the onset of BP suggests that, rather than being simply an adverse reaction to treatment, gliptin-associated BP should in fact be viewed as a drug-aggravated disease. Only a few studies have described the clinical course of DPP-4i-associated BP. In a study by Benzaquen et al. (53) with a follow-up period of 3–30 months, 95% of patients whose DPP-4i therapy was discontinued achieved partial or complete BP remission, while only seen in 55% of those who continued DPP-4i therapy. The population of a recent French study comprised 108 BP patients 45.3% of whose DPP4-i treatment was stopped, with treatment being continued in the remaining 54.7%. The study found no differences between the groups in the median time to achieve disease control, the time to first relapse, the relapse rate, or the mean initial dose of clobetasol propionate cream for the treatment of BP (57). Overall, contradictory findings remain regarding the effect of DPP-4i discontinuation on BP disease course, and further study is required.

Some studies have suggested that DPP-4i-BP occurs more commonly in men and in individuals over 80 years old (53, 56, 58, 67), but not all studies have found such gender or age differences (54, 57). A cohort study from Israel reported that mucous membrane involvement is more likely in DPP-4i-associated BP than in patients without prior DPP-4i exposure (56). Although some distinct clinical features have been reported in cases of gliptin-associated BP, most European studies have concluded that there are no major clinical and immunological differences between DPP-4i-BP and non-DPP-4i-DP (57, 69).

**Figure 3:**
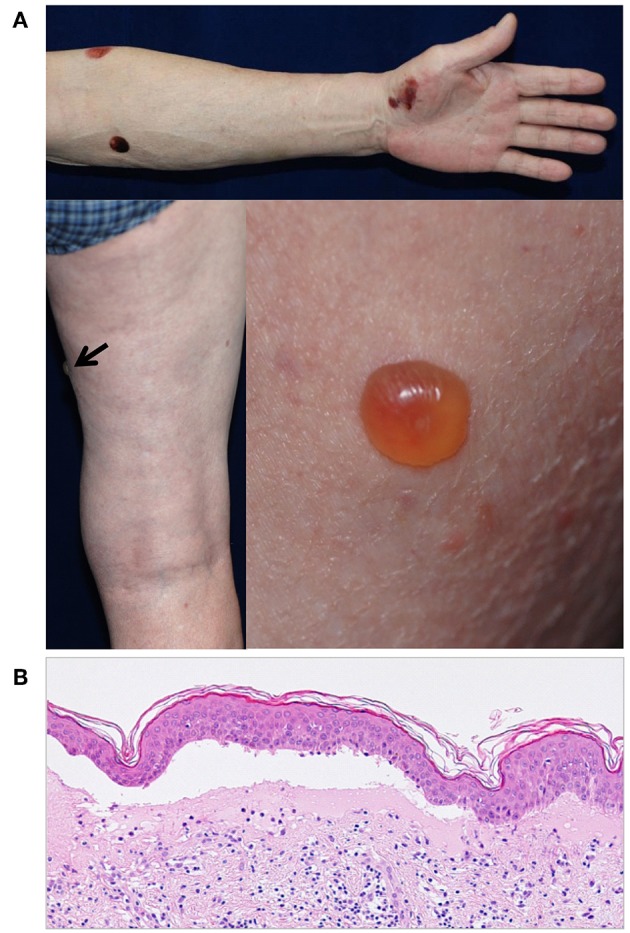
**(A)** A case of DPP4i-BP in a 70-year-old man without autoantibodies directed against the NC16A domain of BP180. The patient had IgG autoantibodies against the non-NC16A domains of BP180. Note that there is no erythema around the blisters. **(B)** The histopathological analysis of lesional skin shows subepidermal blisters with low number of infiltrating eosinophils.

Interestingly, certain Japanese DPP4i-associated BP patients do have distinct clinical and immunological features (70). Firstly, DPP-4i-associated BP in Japanese patients tends to show a non-inflammatory phenotype with less erythema than that seen in typical BP (71) (Figure 3). The low level of inflammation is associated with low numbers of lesional infiltrated eosinophils (70). Secondly, around 40–70% of Japanese patients with DPP-4i-associated BP have a negative or low titer for anti-BP180 NC16A autoantibodies (70–72). This is a sharp contrast to the 80 to 90% of non-DPP-4i-associated BP cases, who are positive for anti-BP180 NC16A autoantibodies (73). In Japanese DPP-4i-associated BP cases, autoantibodies mainly target the mid-portion of the non-NC16A extracellular domain of BP180. It is therefore impossible to detect such autoantibodies using a commercially available kit (70). Similarly to some European cohorts, aged males feature prominently among the Japanese DPP-4i-associated BP population (68), but any elevated likelihood of mucous membrane involvement is still uncertain. However, it should be noted that not all Japanese DPP-4i-associated BP patients show such distinct features, and a cohort study from Kurume University reported that an investigation of BP patients who tested negative for anti-BP180 NC16A autoantibodies revealed no association with prior DPP-4i use (74). Future studies may reveal why distinct clinical and immunological features are observed in some Japanese cases of DPP-4i-associated BP.

### Genetic Characteristics of DPP-4i-Associated BP

The unique clinical and immunological characteristics seen in some Japanese patients with DPP4i-associated BP poses the question as to whether these patients have distinct genetic characteristics, such as particular human leukocyte antigen (HLA) haplotypes. This question was recently addressed by Ujiie et al. (72), in a study on 30 Japanese patients with DPP4i-associated BP. Based on their BP Disease Area Index (BPDAI) scores (75), the patients were divided into two groups: inflammatory, and non-inflammatory. Most patients (21/30) showed the non-inflammatory phenotype and low levels of anti-NC16A BP180 autoantibodies. Interestingly, 86% (18/21) of the non-inflammatory patients had the HLA-DQB1^*^03:01 haplotype, whereas that allele was present in only 31% (19/61) of the diabetes patients receiving DPP-4i without any blister formations. The results clearly suggest that HLA-DQB1^*^03:01 in non-inflammatory DPP-4i-BP is strongly associated with drug-related autoimmune disease (72). Although this study was performed on Japanese BP patients, HLA-DQB1^*^03:01 is also known to be associated with mucous membrane pemphigoid in Caucasian patients (76). Mucous membrane pemphigoid target non-NC16A extracellular domains of BP180 (77), as do DPP4i-associated BP autoantibodies. Therefore, it would be particularly interesting to determine whether HLA-DQB1^*^03:01 is also linked to non-inflammatory DPP4i-associated BP in Caucasian patients. However, a very recent study from Finland failed to detect such an association (52).

### Epitope-Spreading Phenomena in DPP-4i-Associated BP

Models of BP have demonstrated important roles of anti-BP180 NC16A autoantibodies in blister formation (6, 7), while possible pathogenic roles of other autoantibodies targeting the non-NC16A regions of BP180 and BP230 have yet to be identified. However, the presence in DPP-4-associated BP of autoantibodies that target the non-NC16A regions of BP180 suggests that such antibodies may indeed also be pathogenic. Furthermore, anti-BP180 NC16A autoantibodies may not play a primary role in the pathogenesis of DPP-4i-associated BP. A study of four DPP-4i-associated BP patients whose clinical manifestations showed them to have the inflammatory phenotype found that these patients already had polyclonal IgG and IgE autoantibodies targeting various regions of BP180 including the NC16A and intracellular domains, as well as non-NC16A extracellular regions (51). These observations led the notion that the activity of anti-BP180 NC16A autoantibodies may be a secondary rather than a primary phenomenon, arising from epitope-spreading. Recently, several cases of DPP-4i-associated BP have been reported, in which anti-BP180 NC16A autoantibodies developed during the course of the disease (78–80). Interestingly, these case may initially have shown the non-inflammatory phenotype typically associated with autoantibodies directed against the non-NC16A domain of BP180, but their clinical characteristics later changed to a profile consistent with that of the inflammatory phenotype. Furthermore, cessation of DPP-4i treatment did not arrest disease progression, suggesting that the treatment may have triggered the disease, which was then set on a drug-independent course (79).

### Pathogenesis of DPP-4i-Associated BP

Currently the pathogenesis of DPP-4i-associated BP remains largely unclear, but it may be reasonable to expect that the inhibition of CD26 expression on T-cells may have certain effects on the immune system. A murine model showed that inhibition of DPP-4 induces the infiltration of eosinophils into the skin (81). This is notable because the cutaneous infiltration of eosinophils is a typical histopathologic feature of BP. However, the numbers of infiltrating eosinophils in perilesional skin are somewhat lower in non-inflammatory Japanese DPP-4i-associated BP than in cases with the inflammatory phenotype (70, 72).

The major BP autoantigen BP180 is a transmembrane collagen; therefore, certain effects of DPP-4i on the biological activity or metabolism of BP180 may be involved in breaking immunotolerance of BP180. However, a very recent study found no significant effects of DPP4i on the expression or shedding of BP180 in keratinocytes *in vitro* (52). It should also be noted that DPP-4 is a cell-surface plasminogen receptor that converts plasminogen to plasmin, a major serine protease (82). Plasmin is known to cleave BP180 into its 120 and 97 kD ectodomains (83, 84). Therefore, the suppression of DPP-4 may be associated with the development of epitopes for DPP-4i-BP autoantibodies, a possibility that needs to be elucidated by future studies.

It is mysterious that immunotolerance to BP180 is selectively broken in certain individuals by DPP-4i exposure, especially in HLA-DQB1^*^03:01 Japanese carriers (72). It is also curious why BP180 is the only autoantigen for any autoimmune blistering diseases that is targeted by autoimmunity. Although a case of pemphigus vulgaris developing 6 months after the treatment with sitagliptin has been reported (85), BP accounts for the majority of cases of autoimmune blistering diseases likely associated with DPP-4i treatments. Autoimmune blistering diseases, particularly BP, are disproportionately common in DM patients treated with a DPP-4i.

There are several hypothetical mechanisms that may be involved in the breakdown of immunotolerance to BP180, including aberrant expression of BP180 associated with abnormal post-translational modifications or homeostasis. Regarding with this notion, a recent study reported that saxagliptin and sitagliptin both induced epithelial-mesenchymal transition (EMT) in immortalized human keratinocytes (HaCaT), which is associated with increased migration and accelerated wound closure (86). BP180 is known to be involved in keratinocyte migration (87, 88), and EMT may exert an effect on various basement membrane proteins including BP180 (89). Thus, it is possible that DPP-4 inhibition influences keratinocytes in an EMT-dependent manner. It has also been reported that *in vitro* suppression of DPP-4-like activity in fibroblasts inhibits the TGF-β-dependent proliferation of fibroblasts and the secretion of type I procollagen (90).

The final question is whether DPP-4i medication alone is sufficient to induce BP or if other factors are also required. It is currently unknown whether concomitant autoimmune diseases increase the BP risk of BP in gliptin-treated diabetics, since published studies have not yet compared the comorbidities of DPP4i-associated BP patients with those of non-DPP-4i-BP patients. In BP, various triggering factors other than drugs have been reported; including infections, ultraviolet exposure and physical factors such as burns (91). Interestingly, there exists a report of BP having been induced by a thermal burn in a patient who was receiving DPP-4i treatment. In this case, cessation of the DPP-4i led to remission without the use of a systemic corticosteroid (92). The report suggests that DPP-4i treatment may increase the risk of BP but in this case did not independently induce the disease. The results of a French cohort study also suggested that DPP-4i treatment may trigger, but not induce BP (57).

## Future Prospects

Despite the rapidly growing volume of papers regarding the association between the use of gliptins and BP, experimental and clinical data remain scarce. It remains to be seen whether the use of gliptins has any influence on the development of blistering or its severity in BP mouse models. Existing BP models are probably not suitable for this purpose because of the duration of gliptin use required to promote the breakage of immunological tolerance to BP180. On the other hand, we predict that the careful clinical, immunological and genetic characterization of patients with DPP-4i-associated BP will offer valuable information concerning autoimmunity against BP180 and may lead to the development of better treatments for all patients with BP (93).

## Conclusions

Recently published epidemiological data have confirmed firmly that gliptin treatment is a major factor in BP pathogenesis in patients from different ethnic backgrounds. The long latency period between the initiation of gliptin treatment and the beginning or diagnosis of BP suggests that DPP-4i-associated BP is a drug-aggravated, rather than a drug-induced, skin disorder. However, the pathomechanism behind this interesting phenomenon is currently unknown and it remains to be investigated whether gliptins predispose elderly individuals to BP by, for example, disturbing the balance of the immune system and/or altering the structures of the cutaneous basement membrane zone. We need more information concerning the phenotype and prognosis of gliptin-associated BP, and especially concerning the necessity of replacing gliptins with other DM medications in patients with BP. Finally, we sincerely believe that further characterization of gliptin-associated BP will improve our understanding of autoimmunity in general.

## Author Contributions

All authors listed have made a substantial, direct and intellectual contribution to the work, and approved it for publication.

### Conflict of Interest Statement

The authors declare that the research was conducted in the absence of any commercial or financial relationships that could be construed as a potential conflict of interest.
